# Measurement of Spray Chamber Ignition Delay and Cetane Numbers for Aviation Turbine Fuels

**DOI:** 10.1021/acs.energyfuels.5c01350

**Published:** 2025-05-26

**Authors:** Jon Luecke, Nimal Naser, Zhibin Yang, Joshua Heyne, Robert L. McCormick

**Affiliations:** † 53405 National Renewable Energy Laboratory, Golden, Colorado 80401, United States; ‡ Bioproduct, Sciences, and Engineering Laboratory, School of Engineering and Applied Science, 6760 Washington State University, Richland, Washington 99352, United States

## Abstract

Experiments using pure compounds, National Jet Fuels Combustion Program (NJFCP) test fuels, and commercial jet fuels were conducted to demonstrate the equivalence of the indicated cetane number (ICN) and derived cetane number (DCN) for jet fuels. The calibrated range for ICN was also extended to lower cetane number (CN) values (5 to 35) to allow CN quantification for jet fuel synthetic blending components (SBCs) with low CN. ICN and DCN were shown to be highly correlated for values above about 30. This study presents the most comprehensive comparison of these two methods published to date. Because of the importance of low-volume test methods for early-stage SBC production process development, we demonstrated that ICN and DCN can be accurately measured with 15 mL of fuel, well below 40 to 100 mL required by standard methods. ICN or DCN is important for jet fuels because fuels with lower CN are more prone to lean blowout (LBO), an undesirable operational failure in a jet engine. Comparing data on a fuel-to-air ratio (Φ) at LBO for the NJFCP fuels shows similar linear correlations for ICN and DCN. Ignition delay measurements at lower-pressure and higher-temperature conditions may be more directly relevant to LBO. At 675 °C, 0.5 MPa, and a global Φ of roughly 0.68, ignition delay time correlations to LBO were similar to those produced from DCN and ICN. A much weaker correlation was obtained with a global Φ value of 0.34.

## Graphical abstract

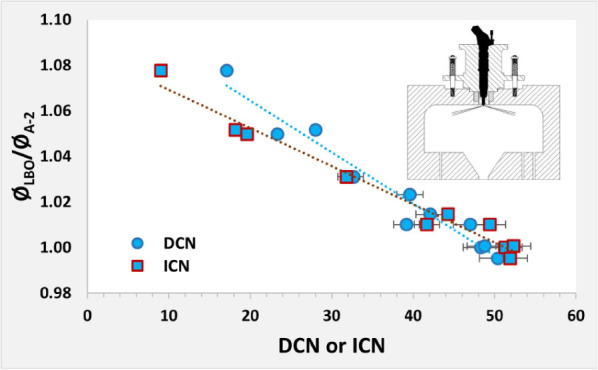

## 1 Introduction

The aviation industry is entering a new era in which petroleum is no longer the sole feedstock for aviation fuels. Emerging technologies that aim to increase the jet fuel supply and reduce pollutant emissions are being actively developed and commercialized. To expand feedstock options, nontraditional materials such as waste plastics, used tires, carbon oxides, and biomass are being explored, offering potential solutions for managing environmental challenges. However, to accelerate commercialization and reduce costs, innovative low-volume test methods are essential. These so-called prescreening methods^1^ can streamline the development process by minimizing the material and financial investment required for ASTM qualification,^2^ as entering this process currently demands approximately $100 million to produce the first hundred gallons for ASTM D4054 qualification.^3^ By enabling cost-effective and efficient evaluation of novel feedstocks and conversion technologies at a much smaller scale, such tools can attract investment and drive novel aviation fuel technologies toward widespread adoption.

At the early stages of developing novel aviation fuel candidates, low-volume testing plays a critical role in guiding producers by illuminating their target properties and acceptable limits. These insights help ensure that novel conversion technologies can efficiently navigate the qualification process. Many of these target properties were developed and refined under the National Jet Fuels Combustion Program (NJFCP), an extensive collaboration that included roughly 40 institutions worldwide.^4^ The NJFCP studied a diverse array of fuels across various thermodynamic conditions and geometries to establish a foundational blueprint for prescreening synthetic aviation turbine fuel (SATF) candidates.^5^

The program emphasized low-volume testing methodologies, particularly figures of merit such as density, viscosity, surface tension, distillation curve, and hydrocarbon-type composition, which were shown to significantly influence a fuel’s ignition and flame stability under critical conditions. Among the three figures of merit studied in detail, one novel finding was the strong correlation between a fuel’s derived cetane number (DCN) and its operability limits in aircraft. The DCN, as measured by ASTM D6890, was found to predict lean blowout (LBO) performance in the referee combustor rig at the Air Force Research Laboratory (AFRL) and other combustor geometries.^6,7^ While DCN is widely employed in research laboratories, the inclusion of additional ignition quality metrics would enhance the global adoption of low-volume testing tools, accelerating the development and deployment of novel SATF technologies.

DCN is an alternative measurement of diesel fuel ignition quality, measured in a constant-volume combustion chamber (CVCC) known as the ignition quality tester (IQT).^8–10^ IQT ignition delay values for diesel fuels under specific conditions are highly correlated with the cetane number (CN) measured in the standard Cooperative Fuel Research engine (as described in ASTM D613), and this correlation converts IQT ignition delay into DCN.^11^ For diesel fuels, CN and DCN are used as a measure of fuel reactivity for autoignition. As mentioned, the DCN has also been inversely correlated with the normalized fuel-to-air equivalence ratio (Φ)—the fuel-to-air ratio divided by the stoichiometric fuel-to-air ratio—for LBO in aircraft engine combustors under some conditions, with higher values of DCN correlated with desired lower values of Φ_LBO_.^7^ The DCN−Φ_LBO_ correlation may, in part, be related to the mechanism of LBO, in which the flame not only becomes unstable with regions of local extinction but also stabilizes or reignites in regions of local autoignition. The limiting value of Φ is attained when autoignition no longer occurs in line with the fact that CN is a measure of reactivity for autoignition.^12,13^ Notably, at lower temperatures and pressures, Φ_LBO_ can be dominated by fuel physical properties impacting spray atomization and breakup.^14^

DCN is roughly correlated with the negative temperature coefficient region reactivity^15,16^ but strongly correlated with low-temperature heat release and weakly correlated with extinction strain rates.^17^ Based on these observations, the guidelines for the qualification of jet fuel synthetic (i.e., not made from petroleum) blending components (SBCs) via the fast-track process recommend that DCN be above 35 to provide adequate resistance to LBO and to fall within the historical range of conventional jet fuels.^18^ An upper DCN value of 60 is intended to prevent durability issues where the fuel autoignites too close to engine hardware, increasing hardware temperatures to undesirable levels.

Here, we investigate the performance of a second accepted CVCC-based diesel fuel CN measurement technique, indicated cetane number (ICN), as measured using ASTM D8183 in an instrument called the advanced fuel ignition delay analyzer (AFIDA).^19,20^ DCN is measured under 2.14 MPa and 545 °C initial conditions, while ICN is measured at 1.75 MPa and 580 °C initial conditions (start of fuel injection). We have previously characterized both the IQT^21,22^ and AFIDA^23^ experiments extensively to extend the instruments’ capabilities beyond DCN and ICN measurement, with the aim of obtaining ignition delay data used to validate chemical kinetic models. This included understanding the internal geometry and temperature stratification along with injection and mixing physics, ultimately leading to validated computational fluid dynamics models for both devices. While the IQT and AFIDA have similar alternative CN measurement capabilities, the research-grade AFIDA experiment offers some advantages over the IQT experiment for more fundamental studies, including operating at higher injection pressure (faster fuel evaporation and mixing), more precise and software-controllable experimental variables (e.g., injection volume, pressure, and temperature), less temperature stratification, and more precise initial conditions.

The objectives of this paper are to demonstrate the equivalence of DCN–ICN for aviation fuels, extend ICN calibration to a lower CN range as some jet fuel SBCs have low CN, present new results showing that ICN and DCN can be measured using as little as 15 mL of fuel, and examine jet fuel and SBC ignition delay under lower-pressure and higher-temperature conditions that may better simulate LBO scenarios. The data presented represent the most comprehensive comparison of DCN and ICN published to date.

## 2 Materials and Methods

### 2.1 Materials

ICN and DCN were measured for multiple pure hydrocarbon compounds obtained from reagent chemical suppliers in high purity (>99%). SBC candidate 1,4-dimethylcyclooctane (DMCO) was obtained from the U.S. Navy.^24^ Previously published data on pure compounds are also included. A list of pure compounds and their ICN and DCN results, along with data sources if previously published, is available in Table S-1. Samples of the NJFCP test fuels were provided by AFRL at Wright-Patterson Air Force Base, Ohio. These fuels were designed to cover a broad range of fuel properties relevant to aircraft gas turbine engine performance and have been previously described in detail.^25^ The NJFCP fuels evaluated were designated as A-1, A-2, A-3, C-1, C-2, C-3, C-4, F-1, F-2, F-3, *n*-C12, S-1, S-2, and J-1. Their ICN and DCN values are listed in Table S-2. Commercially produced hydroprocessed esters and fatty acids (HEFA) synthetic paraffinic kerosene (SPK), alcohol-to-jet (ATJ) SPK, and two commercial Jet A samples were evaluated neat and as blends. These samples and their ICN and DCN values are given in Table S-3. Three cetane-controlled diesel fuels (POSF 12943, 12944, and 12945) made by Haltermann Solutions were also used for the low-volume DCN study.

### 2.2 DCN Experiment

This experiment is conducted using the parameter set according to ASTM D6890, in which air fills the CVCC to a specified pressure at the target temperature, after which fuel is injected, evaporates, mixes, and ultimately ignites. Key values include charge air pressure set to 2.14 MPa, injection by way of a single-hole S-type-delayed pintle diesel injector (inward opening) operating at 22.5 MPa, and charge air temperature (forwardmost thermocouple) between 515 and 575 °C (545 °C nominal), such that the *n*-heptane ignition delay time (IDT) is nominally 3.78 ms. Primary standard-grade 20.9% oxygen balance nitrogen charge air was purchased from Matheson Gas. The total internal chamber volume is approximately 0.2 L. IDT is defined as the difference between the start of combustion (SOC) and the start of injection (SOI). SOI is defined as the moment the nozzle begins to move, as measured by the nozzle motion sensor. The exact SOC definition is proprietary but appears to use a threshold of approximately a 0.14 MPa pressure increase, although it is not stated in the ASTM method. If SOI is defined as zero, then SOC = IDT. There is an initial flush of the fuel injection system followed by 15 preinjections and 32 recorded injections, which require about 100 mL of fuel and take about 25 min to complete. Calibration is primarily performed using high-purity *n*-heptane to adjust the chamber temperature. Methylcyclohexane is also specified in the methodology as a longer IDT standard. A correlation equation has been established between IQT IDT and CN results obtained via ASTM D613 (cetane engine test method), in which the correlated CN predicted from the IDT is called DCN. The DCN range covered is stated to be 31.5–75.1, based on a data set run using both ASTM D6890 and ASTM D613.

Two different IQT instruments were used in various aspects of this study. A model IQT-LM instrument acquired in 2003 by the National Renewable Energy Laboratory was used for most of the new DCN measurements. A model IQT-LM instrument acquired in 2024 by Washington State University was used for some NJFCP test fuel DCN measurements, as well as to demonstrate that DCN could be accurately measured with 15 mL of fuel.

### 2.3 ICN Experiment

The AFIDA experiment for ICN follows the parameters described in ASTM D8183. These include charge air pressure set to a slightly lower value relative to DCN of 1.75 MPa, a notably higher injection pressure (relative to DCN) of 100 MPa using a Bosch CRI3-18 symmetrical seven-hole piezoelectric diesel fuel injector, and charge air temperature (average of two thermocouples) fixed slightly higher at 580 °C. Identical primary standard-grade 20.9% oxygen balance nitrogen charge air was used. The significantly higher injection pressure increases the speed of evaporation and mixing in AFIDA, which has twice the internal volume of the IQT at about 0.4 L. ICN is calibrated using an instrument-specific calibration based on blends of the original CN primary reference fuels, *n*-hexadecane (CN = 100, 99% minimum purity) and 1-methylnaphthalene (CN = 0, 97% minimum purity), which define the CN scale. For measurements according to ASTM D8183, seven calibration fluids with CN numbers 85, 70, 60, 53, 46, 40, and 35 were obtained from the AFIDA manufacturer (ASG Analytik-Service). The resulting measured duplicate IDTs are used to form a calibration curve relating IDT to ICN with *r* ^2^ > 0.998 (typically >0.9995), covering the ICN range of 35–85. We also obtained lower CN standards with cetane numbers of 5, 10, 15, 20, and 27.5 for extending the ICN range to lower values.

To measure ICN, after preparing and filling a vial with 40 mL of the sample, the instrument flushes the fuel injection system and ultimately runs two preinjections followed by 12 recorded injections, taking around 15 min to complete. SOC is defined differently here, as it is the average of the pressure recovery time (*P*_RT_) and the time to reach a 0.15 MPa pressure increase above the initial conditions (*P*_0.15_), while SOI is the point at which the injector is electronically triggered to open. During every injection event, fuel evaporation begins almost immediately, which is detected by the pressure transducer and indicated by a drop in pressure due to evaporative cooling. *P*_RT_ is defined as the time it takes for the pressure to rise back to the initial pressure condition. As for the IQT, if SOI is defined as time zero, then SOC = IDT.

### 2.4 AFIDA IDT Experiment under Non-ICN Conditions

The research version of the AFIDA used here allows full manual control of experimental parameters such as temperature (725 °C maximum), charge pressure (0.5–4 MPa), injection pressure (120 MPa maximum), injection duration (4 ms maximum), data recording duration (4 s maximum), and the ability to program up to four separate injections with selectable timing. A lower-pressure, higher-temperature experiment was conducted under 675 °C and 0.5 MPa initial conditions, thought to be more relevant for LBO. The experiments were conducted in the same way as ICN measurements, including performing 2 preinjections and 12 recorded main injections; however, these yield an IDT value, not an ICN value. For simplicity, the SOC was chosen to be the point at which the pressure in the chamber exceeds 0.15 MPa.

## 3 Results and Discussion

### 3.1 IQT and AFIDA Pressure Data Comparison including SOC Definition

Figure 1 shows pressure data for a single injection collected during both DCN and ICN measurements for sample NREL Jet A (2023 #1) with an ICN of 49.5 and a DCN of 46.0. Note that the *y*-axis displays the pressure differential compared to the initial conditions of the pressurized chamber. The single-injection pressure trace plotted best represents the average from each device, along with SOI, *P*_RT_ (AFIDA data), *P*_0.15_ (AFIDA), or *P*_0.14_ (IQT), and IDT for both experiments. The AFIDA pressure data are considerably less noisy and show less shot-to-shot variability than the IQT data, such that only 12 repeated injections are necessary to obtain a good statistical average for an ICN run, compared to 32 measurements for a DCN run. As a result, the evaporation, PRT, and heat release profiles are considerably more repeatable. SOI is shown in both figures and is easily discerned as the time when the noise level increases considerably as the injection event begins. The different definitions of IDT are also indicated on each pressure trace, as described in Sections 2.2 and 2.3. Despite the differences in noise levels, both instruments produce repeatable IDT values for the DCN/ICN calculation.

**Figure 1:**
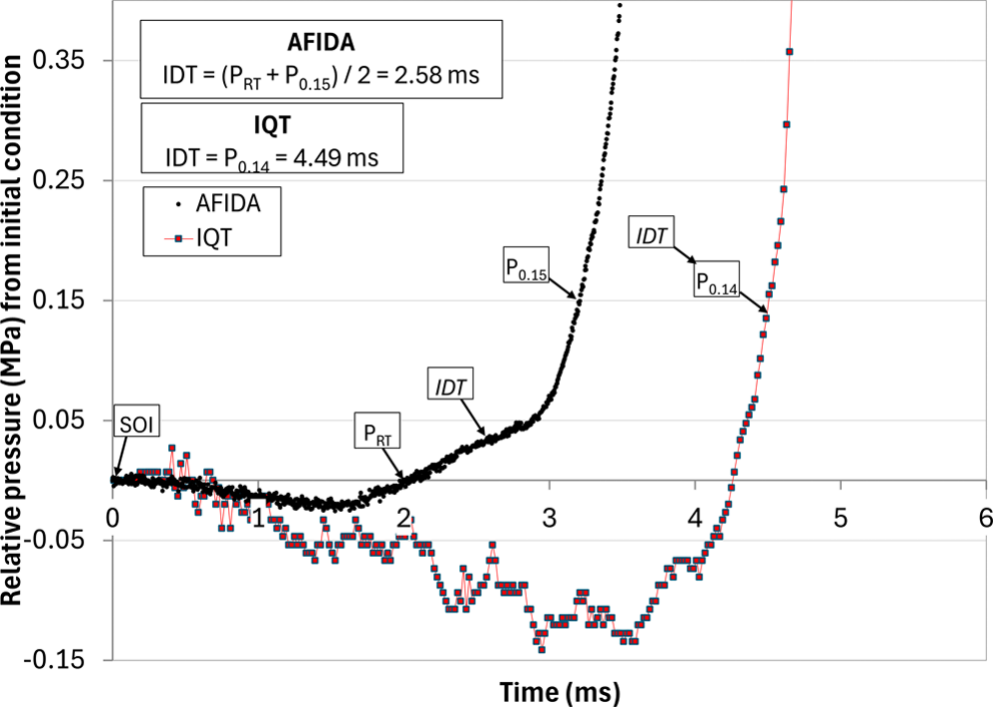
Representative ICN and DCN single-injection pressure trace data for NREL Jet A (2023 #1).

### 3.2 AFIDA Low-CN Calibration

Some potential SBCs have CNs below the calibrated range of both ASTM D6890 and ASTM D8183, such that DCN/ICN values below this range may be less accurate, and ASTM has not developed precision statements for ICN/DCN at these low values. ASTM D8183 is calibrated using blends of primary reference fuels; extending the range requires obtaining new calibration fluids to generate an acceptable IDT-ICN calibration curve. Six low-ICN primary reference fuel blends of *n*-hexadecane/1-methylnaphthalene were obtained with CNs of 5, 10, 15, 20, 27.5, and 35. Because the correlation of ASTM D6890 requires both IQT and Cooperative Fuels Research engine data, extending the range of this experiment requires considerable effort. Figure 2 shows both the standard calibration curve of ASTM D8183 (*r* ^2^ = 0.999, ICN 35 to 85) and the newly generated low-cetane calibration curve (*r* ^2^ = 0.998, ICN 5 to 85). The standard curve works well (<1% absolute error) down to 27.5 ICN, but the error rapidly increases after that point, as indicated in Table S-4. As can be seen from Figure 2, using the standard calibration on a fuel with a lower CN would result in reporting an ICN that is too low by about 6 units for an ICN of 15.

**Figure 2:**
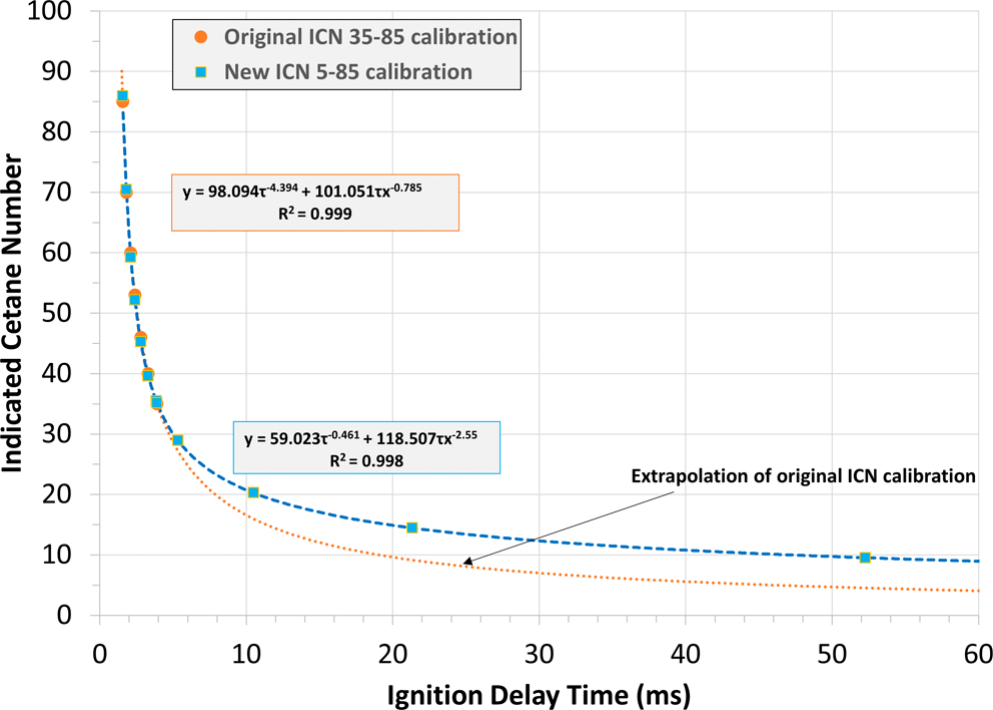
Standard and low ICN calibration curves shown down to an ICN of 10. Instrument-specific equations convert IDT to ICN. Full calibration down to ICN of 5 shown in Figure S-1.

### 3.3 Low-Volume ICN/DCN Validation

ASTM D8183 requires approximately 40 mL of fuel, most of which is used to flush the fuel system thoroughly, ensuring zero carryover from one sample to the next. For early-stage SBC production process development, it is of great interest to be able to assess CN using an even smaller volume of fuel.^1^ A series of experiments with progressively shorter purge times (to minimize the volume requirement) was conducted using two fuels with very different ICN values. The fuel designated as “jet fuel” is Jet A obtained from a commercial jet fuel supplier, with an ICN of 39.7. An SBC (HEFA-SPK) with an ICN of 66.1 was obtained from a commercial fuel producer. The experiment was performed by running the samples back-to-back as the purge time was reduced. Eventually, the previous fuel with a very different ICN will begin to affect results as purging becomes inadequate.

ICN measured with the standard purge time of 420 s was run six times to obtain precise average ICN values for Jet #1 and Jet #2. This yielded average values of 39.7 and 66.1, respectively, with corresponding 95% confidence intervals of ± 0.4 units for jet fuel and ± 1 for HEFA-SPK, compared to ± 0.7 ASTM D8183 repeatability for an ICN of 40 and ± 1.38 for an ICN of 66. Reduction in purge time is plotted on the *x*-axis of Figure 3, in which samples were run twice, each time in succession with HEFA-SPK, which was also run twice. ASTM repeatability of ± 0.7 is reported as the error bars on the lower chart for jet fuel. There is no significant change in measured ICN for jet fuel until the purge time is reduced to 30 s, although the measured ICNs at 50 s purge were both higher than values measured at 100 s and just outside of the measured 95% confidence interval of ± 0.4, indicating there is likely some carryover happening. Reducing the purge time to 100 s is a conservative approach when measuring ICN on samples with small available volume, as carryover is eliminated and volume requirement is reduced to about 15 mL. As further validation, a series of experiments with progressively shorter purge times was also performed with jet fuel and C-1, an ATJ-SPK with an ICN of 8.4. A total of eight ICN determinations were made as the purge time was decreased to 100 s (Figure S2). The average ICN was 8.39 with a 95% confidence interval of ± 0.05.

**Figure 3:**
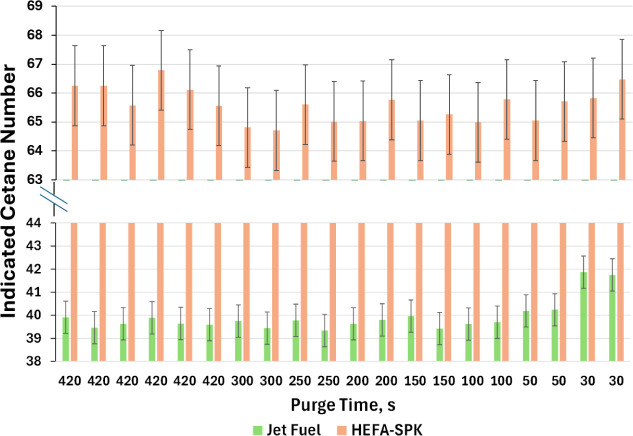
Jet fuel and HEFA-SPK ICN as a function of the shortened purge time. Full sequence of experiments shown going from left to right.

Note that our recommendation for reducing purge time to 100 s for ICN measurements for low-volume samples is specific to hydrocarbon fuels and, to some extent, to fuels within the ICN range examined in these experiments. If a very high-CN fuel is tested, such as a diesel-boiling-range ether with CN ≈ 100,^26^ we recommend running conventional jet fuel or *n*-heptane to fully purge the system before attempting a small-volume ICN. Also, note that this low-volume procedure was developed to be used for preliminary fuel screening, not for fuel qualification or for demonstrating that the fuel meets an ASTM or other standard.

As also shown in Figure 3, the high-cetane jet fuel did not show any significant change in ICN results from any of the reduced purge times, which may indicate that carryover is more sensitive when transitioning from a high-cetane fuel to a low-cetane fuel. This makes intuitive sense, as the low-cetane fuel is relatively less reactive and exhibits a longer IDT, so any highly reactive trace components will be the first to ignite and have a clear influence on IDT, acting like a “cetane improver” even at low levels. The higher-cetane fuel already exhibits a much faster IDT, so, as we show here, trace hydrocarbon components with longer IDTs do not inhibit the initial combustion kinetics.

ASTM D6890 historically required approximately 100 to 150 mL of the sample to complete a DCN test, primarily to ensure thorough flushing of the fuel system and eliminate carryover. However, improvements made by the manufacturer to the newer IQT at Washington State University, such as reducing internal leakage in the pump drain line, have allowed for a smaller fuel reservoir. With these advancements, the actual test procedure comprising 15 preinjections and 32 recorded injections now requires only about 12 mL of the sample.

In this study, a modified procedure was developed to further minimize sample consumption. The modified procedure follows the original ASTM D6890 methodology but replaces the fuel system flushing step with heptane, which is pushed through the system before nitrogen purging for at least 3 min to ensure complete evaporation. After this step, 15 mL of the test sample is introduced into the fuel reservoir, and the standard ASTM D6890 test sequence resumes. The 15 preinjections also help purge any residual heptane from the system.

As shown in Figure 4, the low-volume procedure yields DCN measurements for all NJFCP test fuels and cetane-controlled diesel fuels that fall within the reproducibility limits of published results,^5^ thereby validating the modified procedure. For the SBC candidate DMCO, ASTM D6890 does not report DCN reproducibility for values below 32. The DCN measurement for neat dodecane falls slightly outside the reproducibility range, which may be attributed to the impurity of the neat compounds for the two test results compared to the plot.

**Figure 4:**
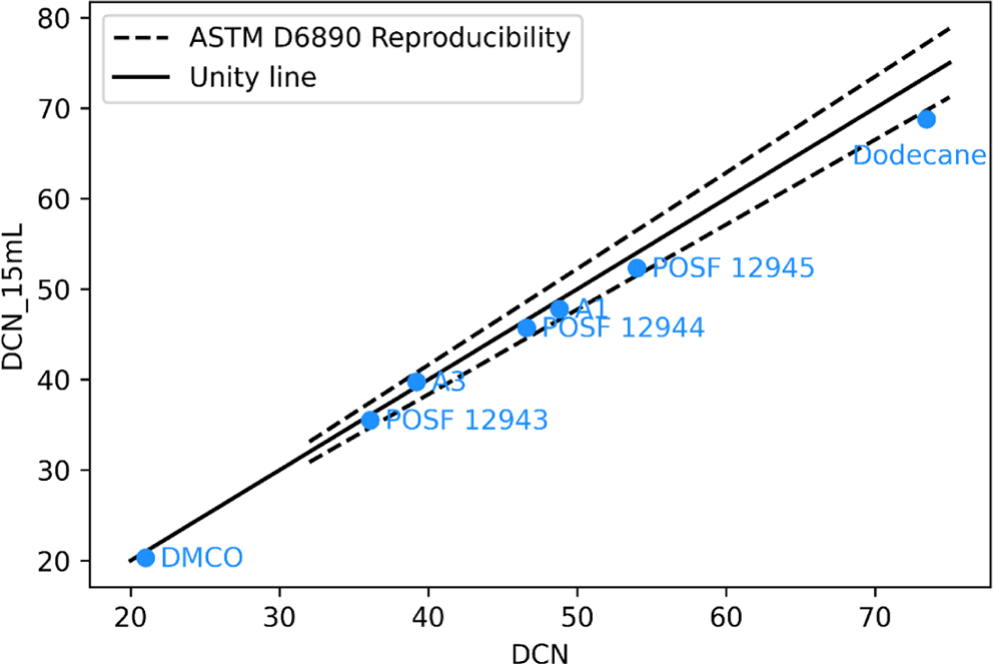
DCN measurements using the low-volume procedure compared to published results for NJFCP test fuels, cetane-controlled diesel fuels, an SATF candidate, and a neat compound.

### 3.4 DCN–ICN Comparison

Figure 5 compares ICN and DCN for pure compounds covering the CN range and carbon types that are common in jet fuels (*n*-alkanes, isoalkanes, and cycloalkanes; aromatics tend to have very low CN and were not included). Above a CN value of about 30 (generally within or nearly within the calibrated range of the methods), agreement between the methods is quite good for all compound classes. Below about 30, values of DCN are consistently higher than values of ICN. Studies comparing CN by ASTM D613 with DCN for diesel fuels, jet fuels, and jet fuel blending components have shown that DCN may overestimate CN below its calibration range (below 32) by 4–6 units.^27,28^ Because ICN values are lower, they may provide a better estimate of ASTM D613 CN for values in this low-CN range.

**Figure 5:**
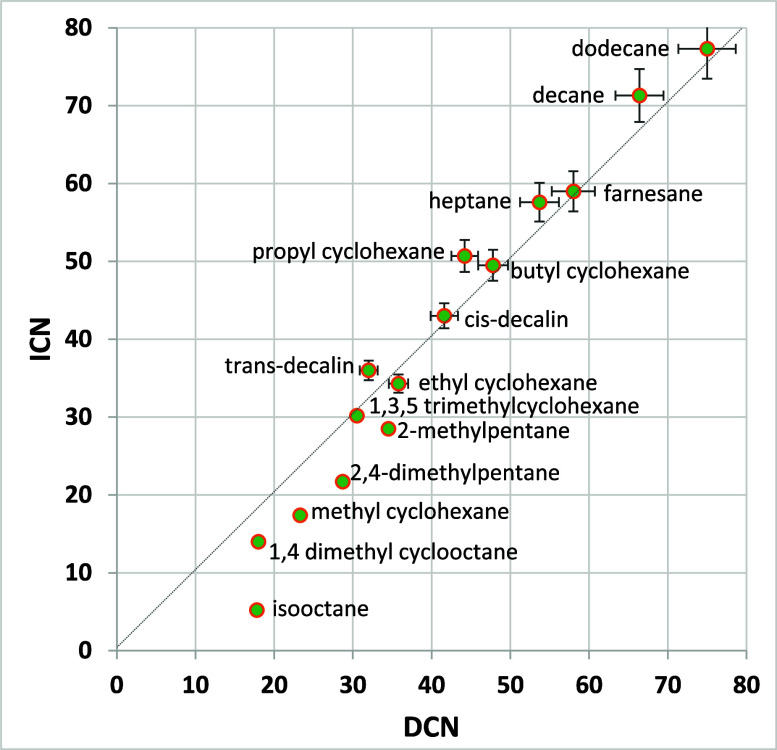
ICN and DCN results for the pure compounds. Error bars are plus or minus ASTM method reproducibility. Below 35 ICN and 32 DCN, precision has not been defined. For ICN, the extended ICN calibration developed in this paper was used.

Figure 6 compares ICN and DCN for various jet fuels (results are tabulated in Tables S-2 and S-3). Examining the results for the NJFCP test fuels shows good agreement above a CN of about 30 but with DCN yielding higher values below about 30. As noted for Figure 5, DCN may overestimate CN below its calibration range (below 30).^27,28^ It seems likely that if the DCN method were calibrated to lower CN, then agreement could be improved. Because DCN is based on a correlation between the IQT ignition delay and ASTM D613 CN data, developing a low-CN calibration requires cetane engine data. While not impossible, this is much more involved than calibrating the AFIDA with primary reference fuels.

**Figure 6:**
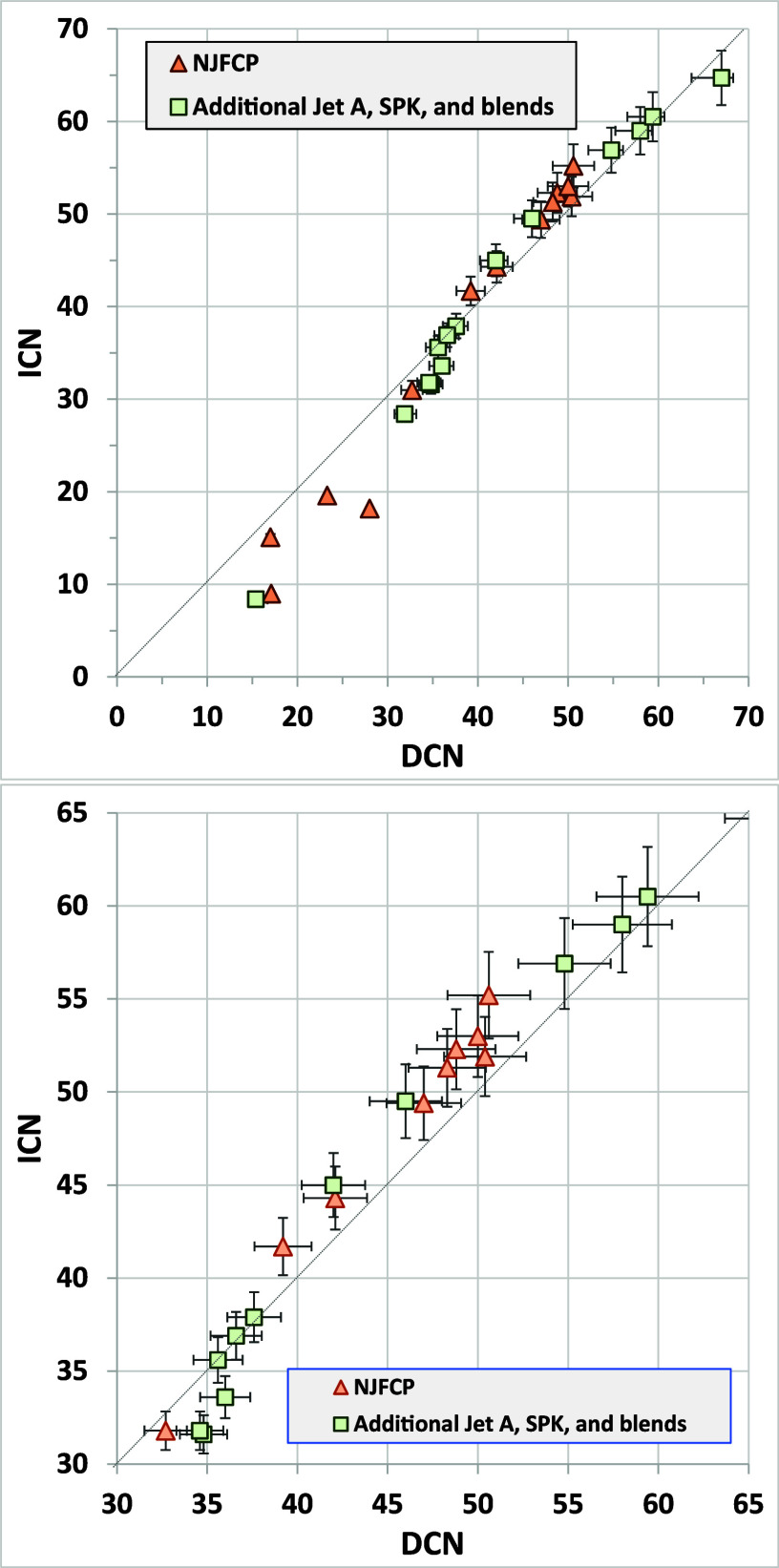
Comparison of ICN and DCN for jet fuel samples; bottom panel zooms in on the 30 to 65 range most relevant for qualification of new SBCs. Error bars are plus or minus ASTM method reproducibility. Below 35 ICN and 32 DCN, precision has not been defined.

### 3.5 ICN/DCN Comparison to LBO Data

Figure 7 shows the comparison of Φ_LBO_ from the AFRL referee rig with DCN for the NJFCP test fuels.^6^ The correlation is highly linear, as previously reported. Eight out of nine LBO experiments (different combustion rigs) in the NJFCP showed a strong dependence of LBO on DCN;^29^ the AFRL referee rig results are shown as an illustrative example. The figure also compares Ø_LBO_ with the ICN measured for the same fuels. The correlation is also highly linear but with a lower slope. Both CN metrics give similar predictions for Φ_LBO_ for CN above 30. Below 30, the overestimation of CN by DCN leads to a minor difference in results. If the DCN method were calibrated below 32, the DCN values for fuels with DCN < 32 could be lower, leading to better agreement between DCN and ICN for the correlations with Φ_LBO_. The difference in predicted Φ_LBO_ for DCN and ICN is within the measurement error for DCN/ICN measurements between 30 and 52. These results strongly support the use of ICN and DCN interchangeably for predicting jet fuel LBO performance for cetane values >30.

**Figure 7:**
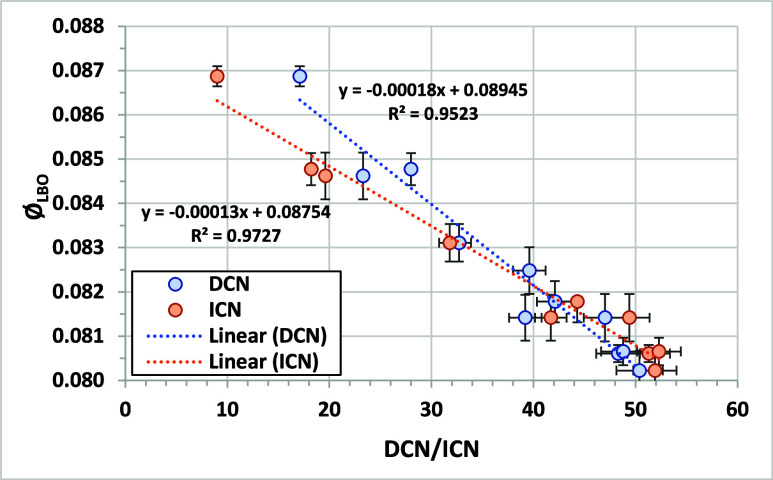
Comparison of Φ_LBO_ in the AFRL referee rig to DCN values for the NJFCP test fuels. Φ_LBO_ data from.^6^ Error bars on DCN or ICN are plus or minus ASTM method reproducibility. Error bars on Φ_LBO_ are plus or minus the 95% confidence interval reported with the results.

Figure 8 shows the same data but also includes two NJFCP surrogate mixtures that have demonstrated preferential vaporization in LBO studies (combustion surrogates are mixtures of a few compounds that mimic or approximate the behavior of more complex fuels).^30,31^ Preferential vaporization, or fractionation, occurs for mixtures when the more volatile (lower-boiling-point) components evaporate first. It can impact LBO when the early-evaporating components have a much lower reactivity (measured as DCN or ICN) than the whole mixture. For surrogates S-1 and S-2, the most volatile components are iso-octane (ICN = 5.2) and 1,3,5-trimethylbenzene (ICN < 5), both of which have much lower autoignition reactivity than the third component (*n*-dodecane with ICN = 77.3 for S-1 and *n*-hexadecane with ICN = 100 for S-2). Both surrogates have ICN/DCN in the 50 to 55 range. However, S-1 impacts LBO as if its CN was about 40, and S-2 as if its CN was about 20. Similarly, in the Georgia Institute of Technology combustor, S-1 and S-2 acted as if their DCNs were 28.7 and 19.1, respectively.^32^ Stachler et al. reported LBO results in a toroidal jet-stirred reactor where reactants were premixed and prevaporized such that preferential vaporization cannot occur.^33^ As shown in Figure 9, both DCN and ICN strongly correlated with Φ_LBO_, and fuel S-1 with an ICN/DCN of 50 to 53 falls right on the correlation line. Our results indicate that preferential vaporization is not occurring in the AFIDA at ICN measurement temperature and pressure (nor in the IQT at DCN conditions) and that measured ICNs reflect the reactivity of the entire fuel mixture.

**Figure 8:**
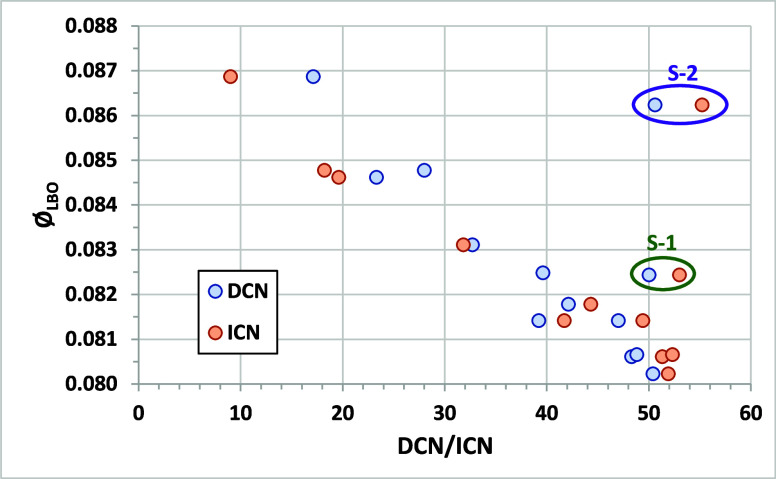
Comparison of Φ_LBO_ in the AFRL referee rig to DCN and ICN values for the NJFCP test fuels and three-component surrogates S-1 and S-2. Φ_LBO_data from.^6^

**Figure 9:**
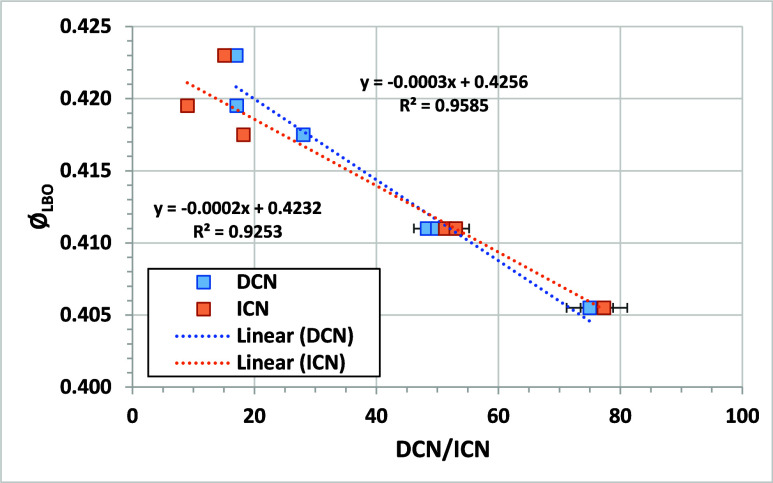
Comparison of Φ_LBO_ in the Stachler et al. toroidal jet-stirred reactor^33^ to DCN and ICN values for the NJFCP test fuels and three**-**component surrogate S-1. Correlation to DCN previously shown by Stachler et al.

### 3.6 Correlation of AFIDA IDT Results at Higher Temperature and Lower Pressure with Φ_LBO_

Previous NJFCP data correlating Φ_LBO_ with DCN (and ICN, as we have now shown) look promising, but it could be beneficial for ongoing investigations to examine IDT under conditions that are more relevant to LBO and are not constrained by comparison to cetane engine results or cetane primary reference fuels. The NJFCP experiments to measure Φ_LBO_ were conducted at pressures ranging from 0.1 to 0.9 MPa;^29^ we have measured IDT at the lower-limit initial pressure for the AFIDA of 0.5 MPa. While the temperature for LBO is more difficult to determine given the sensitivity of DCN/ICN to the negative temperature coefficient region,^15,16^ our selected temperature is at roughly the transition between high-temperature combustion and the negative temperature coefficient region at 675 °C. We also employed two different global Φ values: a leaner condition that used an injection duration of 500 μs and a richer condition where injection duration was set at 758 μs to deliver twice the fuel volume and produce twice the global Φ. Dodecane global Φ values under the lean and rich conditions are 0.34 and 0.68, respectively, but fuel composition differences will cause Φ to vary between fuels, as it does for both ICN and DCN, as injection duration is held constant. The fuels tested were *n*-dodecane and NJFCP fuels A-1, A-2, A-3, C-1, C-4, S-1, S-2, and J-1, allowing comparison to both the referee rig and toroidal jet-stirred reactor results, as shown in Figure 10.

**Figure 10:**
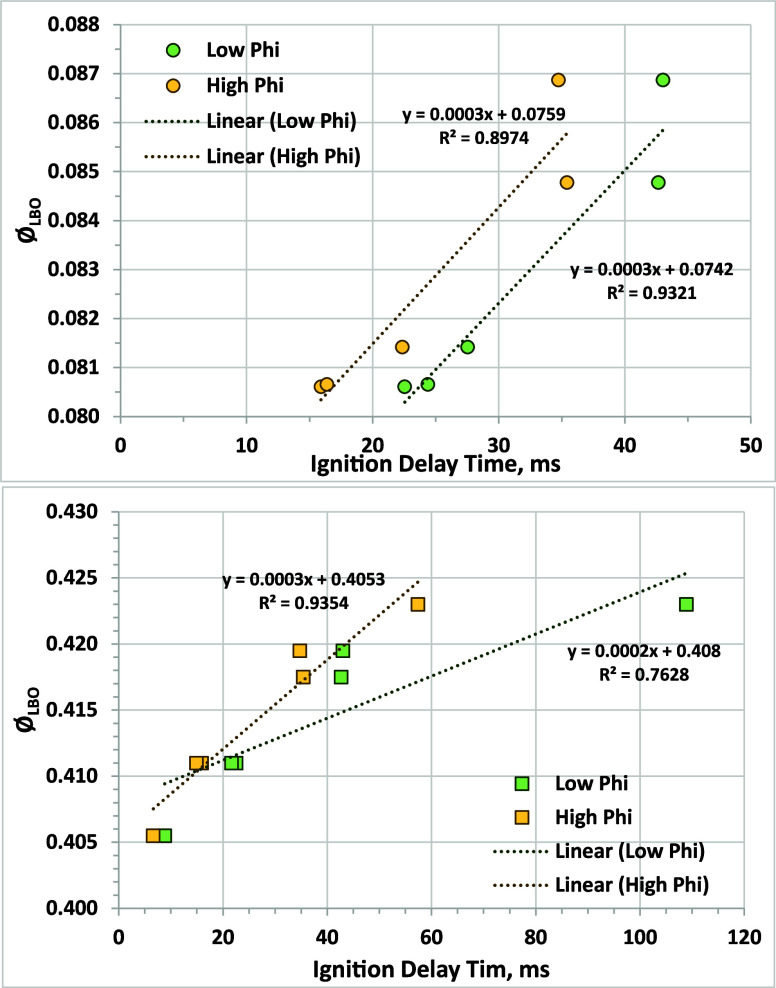
Correlation of AFIDA IDT at 675 °C, 0.5 MPa, and global Φ of nominally 0.34 (low phi) and 0.68 (high phi) with Φ_LBO_results from the AFRL referee rig (top panel, including fuels A-1, A-2, A-3, C-1, and C-4)^6^ and the toroidal jet-stirred reactor (bottom panel, including fuels A-2, C-4, C-4, S-1, S-2, J-1, and dodecane).^33^

For the referee rig, strong correlations between Φ_LBO_ and IDT are observed at both IDT Φ values. For the lowest-ICN fuels (highest-IDT fuels), the results show significantly different Φ_LBO_ values but very similar IDT values. This indicates that the use of DCN or ICN provides better discrimination between fuels at low reactivity levels for LBO in the referee rig. This is also reflected in the higher *r* ^2^ value in Figure 7 versus Figure 10. For the toroidal jet-stirred reactor, a strong correlation between Φ_LBO_ and IDT is observed for the higher Φ value (*r* ^2^ = 0.94). However, at the low value of Φ, *r* ^2^ is much smaller. This is driven by the much higher ignition delay for fuel J-1 at low Φ versus high Φ. For the other fuels, the difference between IDT at low and high Φ is less than 10 ms, but for J-1, this difference is about 50 ms. J-1 was developed to have the same DCN as C-1, a 100% isoparaffinic fuel, but it contains a high level of aromatics (75.5% 1,3,5-trimethylbenzene). Under these lower-pressure and higher-temperature conditions, J-1 is significantly less reactive than C-1 (longer IDT) and shows higher Φ_LBO_ (0.4230 versus 0.4195). This trend toward lower reactivity is significantly worse at the lower Φ value used in these experiments, perhaps pointing to aspects of aromatics autoignition chemistry that are sensitive to Φ. Overall, comparing results in Figures 7 and 9 with those in Figure 10 shows no benefit for measuring ignition delay under these non-ICN conditions, as coefficients of determination (*r* ^2^) are not significantly higher and because of the poor discrimination between low-reactivity fuels based on Φ_LBO_ measured in the referee rig. Nevertheless, additional research to optimize constant volume chamber conditions for the prediction of Φ_LBO_ may yield a useful method that is fundamentally more relevant than the DCN or ICN methods.

## 4 Conclusion

DCN data generated following ASTM D6890 using an IQT device are compared to ICN data following ASTM D8183 using an AFIDA device on fuels used as part of the NJFCP. These fuels had been previously characterized in multiple combustor designs for Φ_LBO_, an important fuel property metric for qualifying new SBCs. Previous research shows a strong inverse correlation between DCN and Φ_LBO_ ^6,7,29,30,33^ and newly generated ICN measurements show a very similar correlation, with a slightly higher *r* ^2^ (0.97 versus 0.95) and a slightly lower slope. These results indicate that ICN is equivalent to DCN for predicting Φ_LBO_ and possibly an improvement due to its more accurate low-cetane calibration.

To reduce the sample volume required for ICN measurement, we investigated reducing the purge time to 100 s from 420 s. Similarly, a change in the purging strategy was also implemented in the IQT to reduce the sample volume required for measuring DCN. These experiments demonstrated that valid ICN or DCN values can be obtained using only 15 mL of sample volume, a significant reduction compared to the 40 mL volume in the standard ICN method or nominally 100 mL for the standard DCN method. This is an obvious advantage for early-stage SBC development, in which volume limitations inhibit fuel characterization.^1^

Potential SBCs already exist that present CNs below the calibrated range of either ASTM D8183 or D6890, resulting in poorly quantified ICN/DCN and raising questions regarding the usability of the data. Using the same methodology employed for the AFIDA standard calibration, recalibration of ASTM D8183 was performed over the range of 5–85 ICN, yielding a new IDT-to-ICN correlation with *r* ^2^ = 0.998, which provides much more accurate low-cetane results than the standard curve, especially below an ICN of about 27.5.

Measurement of IDTs of NJFCP test fuels at 675 °C and 0.5 MPa was undertaken, as this lower pressure and higher temperature relative to ICN measurement may be more relevant to LBO. The experiment was conducted at two Φ values and compared to published Φ_LBO_ values for the AFRL referee rig^6^ and the toroidal jet-stirred reactor **(33)**. Overall, the results show no benefit in measuring ignition delay under these non-ICN conditions, as *r* ^2^ values are not significantly better and because of the poor discrimination of low-reactivity fuels for the referee rig.

## Supplementary Material



## Notes

Tabulated ICN and DCN data, ICN calibrations, and associated errors (PDF)
