# Supplementary material for: Measurement of Spray Chamber Ignition Delay and Cetane Numbers for Aviation Turbine Fuels
Source: Energy Fuels. 2025 May 26;39(22):10479–87. doi: 10.1021/acs.energyfuels.5c01350 (PMC12147154; doi:10.1021/acs.energyfuels.5c01350)
Supplement: Supplementary file 1 [file ef5c01350_si_001.pdf]

Supporting Information for

Measurement of Spray Chamber Ignition Delay and Cetane Number for Aviation Turbine Fuels

Jon Luecke[1], Nimal Naser[1], Zhibin Yang[2], Joshua Heyne[2], Robert L. McCormick[1*]

[1]National Renewable Energy Laboratory, Golden, CO, USA

[2]Bioproduct, Sciences, and Engineering Laboratory, School of Engineering and Applied Science, Washington State University, Richland, WA, USA

Table S-1. ICN and DCN Results for Pure Compounds

Table S-2. ICN and DCN Results for NJFCP Test Fuels

Table S-3. DCN and ICN of Conventional Jet A and Synthetic Blend Component Samples

Figure S-1. Original (standard) and low-ICN calibrations, full range down to ICN of 5

Table S-4. Low-ICN calibration points and associated error

Table S-5. Standard ICN calibration points and associated error including low-ICN PRFs

[*] Corresponding author: robert.mccormick@nrel.gov

Table S-1. ICN and DCN Results for Pure Compounds (low CN calibration used for ICN).

Compounds	DCN	ICN	Data source
heptane	53.7	57.6	ICN this work, DCN [1]
isooctane	17.8	5.2	ICN this work, DCN [1]
1,4 dimethyl cyclooctane	18	14	ICN this study, DCN [2]
trans-Decalin	32	36	ICN this study, DCN [3]
cis-Decalin	41.6	43	ICN this study, DCN [3]
2,6,10 trimethyl dodecane (farnesane)	58	59	ICN [4], DCN [5]
n-decane	66.4	71.3	ICN this study, DCN [1]
n-dodecane	75	77.3	ICN this study, DCN [6]
methyl cyclohexane	23.3	17.4	ICN this study, DCN [1]
butyl cyclohexane	47.8	49.5	ICN [4], DCN [1]
propyl cyclohexane	44.2	50.7	ICN and DCN this study
1,3,5 trimethyl cyclohexane	30.5	30.2	ICN this study, DCN [1]
2-methylpentane	34.5	28.5	ICN this study, DCN [1]
2,4-dimethylpentane	28.7	21.7	ICN this study, DCN [1]
ethyl cyclohexane	35.8	34.3	ICN this study, DCN [1]

Table S-2. ICN and DCN Results for NJFCP Test Fuels (low ICN calibration used)

NJFCP ID	DCN [6]	ICN[a]
A-1 (10264)	48.8	52.3
A-2 (10325)	48.3	51.3
A-3 (10289)	39.2	41.7
C-1 (13718)	17.1	9
C-2 (12223)	50.4	51.9
C-3 (12959)	47	49.4
C-4 (13217)	28	18.2
F-1 (20% C-1 in A-2)	42.1	44.3
F-2 (50% C-1 in A-2)	32.7	31.8
F-3 (80% C-1 in A-2)	23.3	19.6
S-1[b]	50	53
S-2[c]	50.6	55.2
J-1[d]	17	15.1

[a]ICN this work. [b]Surrogate defined in NJFCP, blended at NREL: 59.3 vol% n-dodecane, 18.4 vol% isooctane, 22.2 vol% 1,3,5 trimethylbenzene. [c]Surrogate defined in NJFCP, blended at NREL: 52.6 vol% n-hexadecane, 25.1% isooctane, 22.2% 1,3,5 trimethylbenzene. [d]24.5 vol% n-dodecane, 75.5% 1,3,5 trimethylbenzene.

Table S-3. DCN and ICN of Conventional Jet A and Synthetic Blend Component Samples (low ICN calibration used). Blend percentages in vol%.

	DCN (NREL)	ICN (NREL)	
Commercial HEFA-SPK#1	59.4	60.5	
Commercial HEFA-SPK#2	67	64.7	
Commercial ATJ-SPK	15.4	4	Isobutanol-derived
A-2 + 50% HEFA-SPK#1	54.8	56.9	
NREL Jet A (2023 #1)	46.0	49.5	Commercial Jet A
2023 #1 + 50% ATJ-SPK	34.8	31.6	
2023 #1 + 45% ATJ-SPK	36.0	33.6	
2023 #1 + 35% ATJ-SPK	37.6	37.9	
NREL Jet A 2023 #A	42.0	45.0	Commercial Jet A
2023 #A + 50% ATJ-SPK	31.9	28.4	
2023 #A + 40% ATJ-SPK	34.6	31.8	
2023 #A + 30% ATJ-SPK	35.6	35.6	
2023 #A + 25% ATJ-SPK	36.6	36.9	

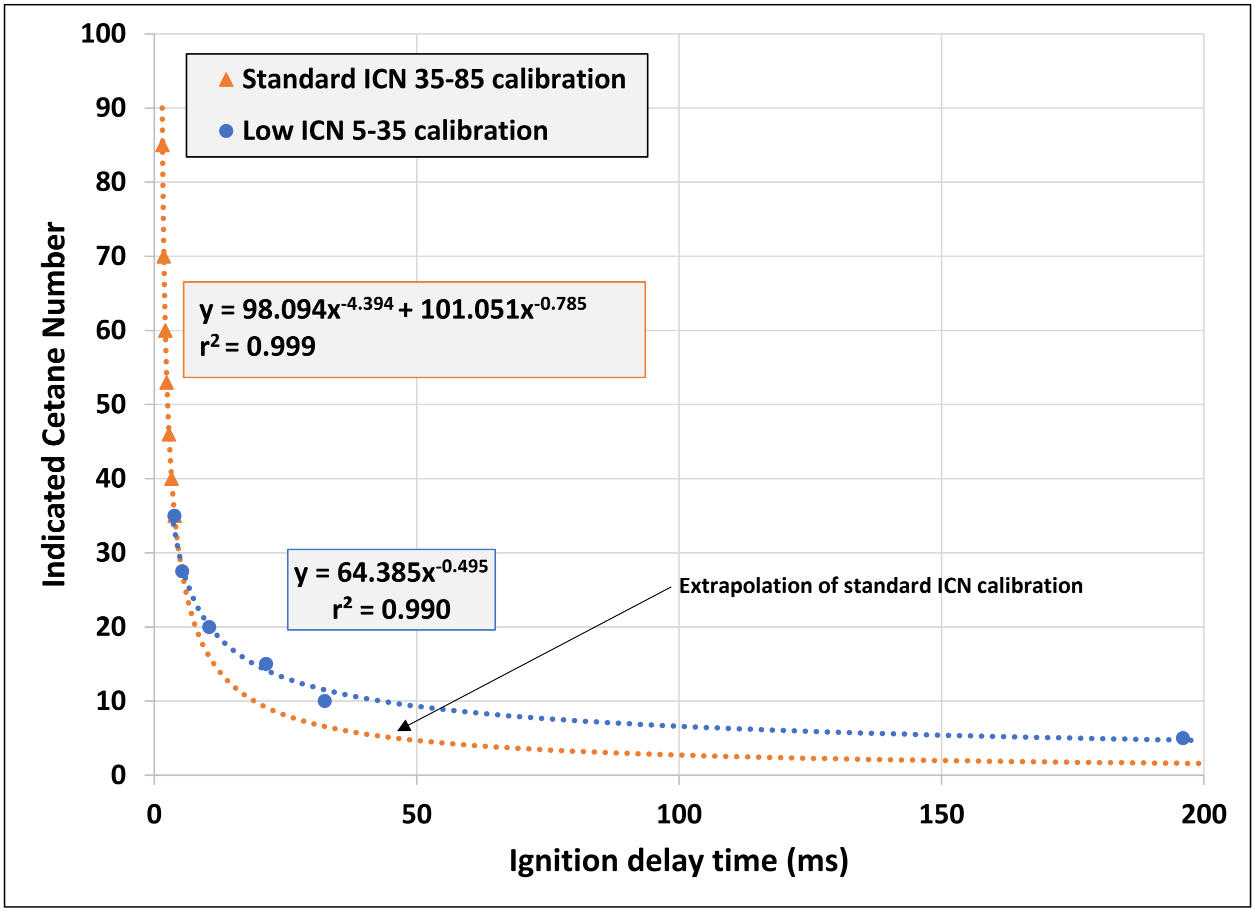

Figure S-1. Original (standard) and low-ICN calibrations, full range down to ICN of 5.

Table S-4. Low-ICN calibration points and associated error

Low ICN calibration (5–35) Data				
**ICN**	**IDT**	**Cal Curve Response**	**Actual unit error**	**Error (percent)**
35.0	3.86	33.03	-1.97	-5.6%
27.5	5.33	28.13	0.63	2.3%
20.0	10.49	20.13	0.13	0.7%
15.0	21.34	14.17	-0.83	-5.5%
10.0	32.49	11.51	1.51	15.1%
5.0	196.05	4.73	-0.27	-5.4%

Table S-5. Standard ICN calibration points and associated error including low-ICN PRFs

Standard ICN calibration (35–85) applied to all PRF data				
**ICN**	**IDT**	**Cal Curve Response**	**Actual unit error**	**Error (percent)**
85.0	1.56	84.97	-0.03	0.0%
70.0	1.82	70.21	0.21	0.3%
60.0	2.12	59.75	-0.25	-0.4%
53.0	2.39	53.03	0.03	0.1%
46.0	2.80	46.14	0.14	0.3%
40.0	3.30	40.08	0.08	0.2%
35.0	3.90	34.94	-0.06	-0.2%
35.0	3.86	35.29	0.29	0.8%
27.5	5.33	27.21	-0.29	-1.0%
20.0	10.49	15.97	-4.03	-20.2%
15.0	21.34	9.14	-5.86	-39.0%
10.0	32.49	6.57	-3.43	-34.3%
5.0	196.05	1.60	-3.40	-67.9%

References

[1] J. Yanowitz, M. Ratcliff, R. McCormick, J. Taylor and M. Murphy, "Compendium of Experimental Ceetane Numbers," National Renewable Energy Laboratory NREL/TP-5400-67585, Golden, CO, 2017.

[2] K. Rosenkoetter, C. Kennedy, P. Chirik and B. Harvey, "[4 + 4]-cycloaddition of isoprene for the production of high-performance bio-based jet fuel," *Green Chemistry,* vol. 21, p. 5616, 2019.

[3] J. Heyne, A. Boehman and S. Kirby, "Autoignition Studies of trans- and cis-Decalin in an Ignition Quality Tester (IQT) and the Development of a High Thermal Stability Unifuel/Single Battlefield Fuel," *Energy and Fuels,* vol. 23, no. 12, pp. 5879-5885, 2009.

[4] G. Fioroni, L. Fouts, J. Luecke, D. Vardon, N. Huq, E. Christensen, X. Huo, T. Alleman, R. McCormick, M. Kass, E. Polikarpov, G. Kukkadapu and R. Whitesides, "Sceening potential biomass-derived streams as fuel blendstocks for mixing controlled compression ignition combustion," *SAE Technical Paper No. 2019-01-0570,* 2019.

[5] T. Smagala, E. Christensen, K. Christison, R. Mohler, E. Gjersing and R. McCormick, "Hydrocarbon Renewable and Synthetic Diesel Fuel Blendstocks: Composition and Properties," *Energy and Fuels,* vol. 27, no. 1, pp. 237-246, 2013.

[6] T. Edwards, "Property Data for Category A and Category C Fuels," in *Fuel Effects on Operability of Aircraft Gas Turbine Combustors*, Reston, VA, American Institute for Aeronautics and Astronautics, 2021, pp. 563-612.
